# Characterization and Comparative Analysis of the *Actinidia rufa* Mitochondrial Genome

**DOI:** 10.3390/genes17101209

**Published:** 2026-09-29

**Authors:** Chengcheng Ling, Zirou Xie, Hang Yuan, Jun Yang

**Affiliations:** College of Food and Bioengineering, Bengbu University, Bengbu 233000, China; lingchengcheng402@163.com (C.L.); 18656228756@163.com (Z.X.); 16655333061@163.com (H.Y.)

**Keywords:** *Actinidia rufa*, mitochondrial reference, dispersed repeats, RNA editing, gene-tree concordance

## Abstract

Background/Objectives: *Actinidia rufa* provides a wild kiwifruit resource for examining mitochondrial sequence conservation and structural variation. We examined the hypothesis that coding-sequence (CDS) coordinates retain higher interspecific alignment coverage than non-CDS coordinates despite differences in block organization. We also assessed reference support, repeat-associated junctions, and mitochondrial gene-tree concordance. Methods: NC_086781.1 was evaluated using PacBio HiFi reads (CRR3429456), uniform repeat-junction screening, six interspecific one-to-one comparisons, repeat and mitochondrial plastid DNA (MTPT) masking, and single-gene trees. Results: The 1,022,459 bp reference contains 39 protein-coding loci. Across six comparisons, CDS coverage was 99.90–100%, versus 68.94–87.79% for non-CDS sequence; adjusted circular-shift *p* values were 0.001674–0.008745. Homologous sequence occurred in 32–61 merged blocks per comparison, and separate repeat and MTPT masks retained 94.39–98.29% and 93.60–99.39% of baseline aligned reference sequence, respectively. Stringent competitive mapping gave a mean depth of 40.35× and breadth of 73.9% at ≥1×. Of 192 repeat pairs ≥50 bp, 41 were evaluable and none met the reciprocal-alternative-support rule; R43 and R47 each had one-sided single-read support. Eight of 16 informative gene trees contained the *A. rufa*–*A. chinensis* split, but phylogenetic information was limited. Conclusions: The comparisons support higher CDS than non-CDS coordinate coverage within a reference set with variable block organization. Repeat-junction support and single-gene resolution remain limited; the data do not establish ongoing recombination, an exclusive circular physical architecture, or a mitochondrial contribution to waterlogging tolerance.

## 1. Introduction

Plant mitochondria sustain oxidative phosphorylation and participate in carbon metabolism, stress responses, programmed cell death, and retrograde signalling [1,2,3,4]. Their genomes are strikingly different from the compact mitochondrial genomes of most animals: land-plant mitogenomes vary greatly in size, chromosome number, gene order, and noncoding content, yet the nucleotide substitution rates of many mitochondrial protein-coding genes remain comparatively low [5,6,7,8,9]. This combination of conserved bioenergetic function and rapidly changing architecture makes plant mitogenomes powerful systems for studying organelle–genome evolution. Recombination and double-strand-break repair are major forces in the maintenance and restructuring of plant mitochondrial DNA [3,10]. Large repeats often undergo frequent homologous recombination, whereas intermediate and short repeats can produce low-abundance alternative molecules whose stoichiometry may vary among tissues, developmental stages, genotypes, or stress conditions [10,11]. Accordingly, physical plant mitochondrial DNA is better viewed as a dynamic population of linear, branched, circular, and subgenomic molecules than as an obligatory master circle [11,12,13]. A circular GenBank sequence is therefore a useful reference representation, but long reads spanning repeats and their flanks are needed to determine which alternative junctions are present in vivo.

Plant mitochondrial genomes are also mosaics shaped by DNA movement among the mitochondrion, plastid, and nucleus [14,15]. Plastid-derived segments may persist in mitochondria as nonfunctional fragments or occasionally contribute genes and tRNAs, while mitochondrial genes themselves have repeatedly been lost or functionally transferred to the nucleus [8,14,15]. In parallel, extensive C-to-U RNA editing modifies many plant mitochondrial transcripts and can restore evolutionarily conserved amino acids, create start or stop codons, and influence respiratory-complex biogenesis [16]. These processes further decouple the information contained in a genomic reference sequence from the mature mitochondrial transcriptome and functional proteome.

The genus *Actinidia* includes cultivated kiwifruits and diverse wild relatives distributed primarily in eastern Asia, but its infrageneric relationships and species boundaries remain complex [17]. *A*. *rufa* is particularly valuable for rootstock improvement and introgressive breeding because of its elite waterlogging tolerance; a chromosome-level nuclear assembly recently linked this trait to root anatomical, hormonal, and transcriptional responses and supported a close relationship with *A. chinensis* [18]. Organelle–genome resources are needed to complement these nuclear data and to trace maternal lineages, cytoplasmic diversity, and structural evolution.

Mitochondrial comparative genomics in *Actinidia* has progressed substantially through our group’s successive studies. Wang et al. first demonstrated that kiwifruit mitogenomes diversified through extensive whole-genome rearrangement and mosaic loss of intergenic sequences [19]. Our subsequent comparison of five Actinidiaceae mitogenomes documented large size differences, extensive repeats, plastid-homologous fragments, RNA-editing patterns, and pronounced rearrangement despite conserved coding capacity [20]. We then produced high-quality assemblies of *A. latifolia* and *A. valvata*, revealing one- versus two-circle reference representations and marked differences in plastid-derived content [21], and extended the same framework to *A. macrosperma*, whose 752,501 bp mitogenome contains abundant dispersed repeats and intracellularly transferred fragments [22]. Together, these studies established that *Actinidia* mitogenomes are structurally dynamic rather than uniformly circular or collinear.

Although several *Actinidia* mitogenomes have been reported, it remains unclear how coding and noncoding sequence retention in *A. rufa* compares with variation in aligned block organization, and which structural interpretations are supported by its HiFi reads. Building on the conserved coding capacity and variable organization reported in *Actinidia* [19,20,21,22], we examined the hypothesis that CDS coordinates retain higher interspecific alignment coverage than non-CDS coordinates despite differences in block organization. Here, conservation refers to the fraction of annotated coordinates recovered in filtered alignments, not conserved gene order, equal nucleotide identity, or a causal evolutionary mechanism. Our objectives were to (1) characterize NC_086781.1 and assess the read support for its coordinates and repeat-associated junctions; (2) quantify block organization and the CDS versus non-CDS coverage contrast across six *Actinidia* comparisons, with separate repeat and MTPT masking analyses to assess the sensitivity of overall alignment coverage; and (3) assess whether the concatenated placement of *A. rufa* is consistently recovered by individual mitochondrial genes. RNA gene annotation checks, codon usage, and predicted RNA editing provide supporting reference characterization. Recombination activity, nuclear control, and waterlogging adaptation are mechanistic questions for future testing, not hypotheses tested by the present dataset.

## 2. Materials and Methods

### 2.1. Plant Material, DNA Extraction, and PacBio HiFi Sequencing

Fresh young leaves were collected on 6 July 2025 from a wild *A. rufa* individual in Desheng Town, Yizhou City, Guangxi Province, China (24.650° N, 108.400° E). The plant material was deposited in the BBU105 Laboratory, College of Food and Bioengineering, Bengbu University. A laboratory reference specimen from the same plant is retained in the BBU300 Laboratory; it has not been accessioned as a formal herbarium voucher. Leaves were wrapped in aluminum foil, immediately frozen in liquid nitrogen, and stored at −80 °C, following the sample-handling approach used in our earlier Actinidiaceae study [20]. High-molecular-weight genomic DNA was isolated with the DNAsecure Plant Kit (Tiangen Biotech, Beijing, China) according to the manufacturer’s instructions. DNA integrity and purity were assessed before library preparation.

A SMRTbell library was sequenced on the PacBio Revio platform (Pacific Biosciences, Menlo Park, CA, USA). The resulting HiFi run comprised 740,136 reads totaling 13,794,039,825 bp (13.79 Gb), with a mean read length of 18,637 bp, median length of 17,615 bp, read N50 of 18,748 bp, and maximum length of 57,759 bp. The raw data are publicly available in the Genome Sequence Archive under BioProject PRJCA071801, accession CRA048345, run CRR3429456. The mean depth of stringently and competitively assigned HiFi alignments across NC_086781.1 was 40.35×.

### 2.2. Mitochondrial Reference Evaluation and Annotation

NC_086781.1 was used as the 1,022,459 bp mitochondrial coordinate reference for annotation and downstream analysis. Its circular representation is not treated as evidence for an exclusive master-circle chromosome. For read-based evaluation, CRR3429456 HiFi reads were competitively aligned against NC_086781.1 and plastome NC_039973.1 using minimap2 v2.26-r1175 in map-hifi mode [23]. Coverage was calculated from primary mitochondrial alignments with mapping quality ≥20 and bases with quality ≥10. A synthetic terminal-to-start reference was screened with 1, 2, and 5 kb flanking anchors for uniquely spanning reads. These checks evaluate support for the reference coordinates; they neither reconstruct a complete physical architecture nor establish that the deposited sequence was independently reproduced by these analyses.

**Figure 1:**
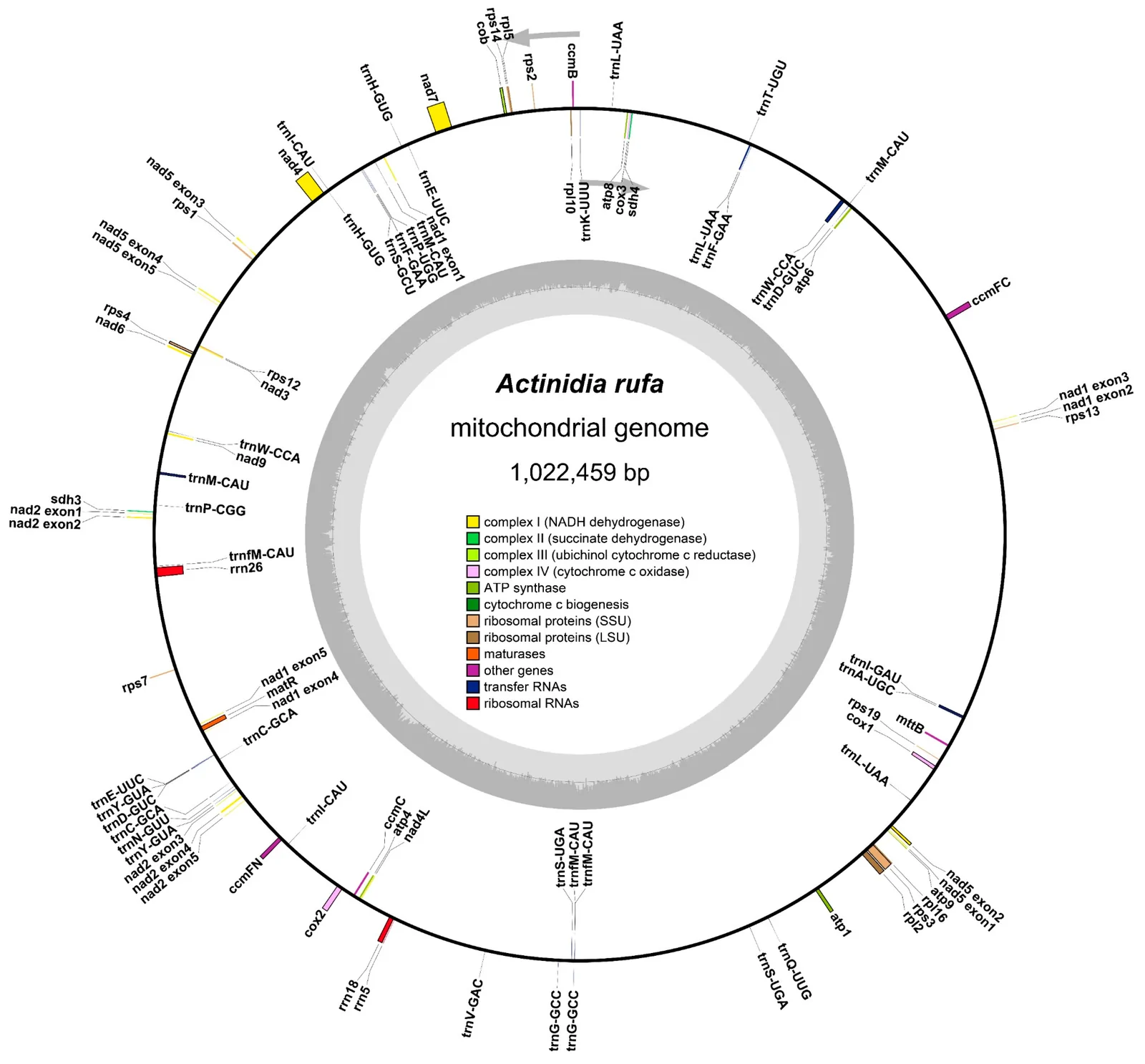
Circular coordinate map of the *A. rufa* mitochondrial reference NC_086781.1. Outer and inner gene labels indicate the two transcriptional orientations. The internal shading shows base-composition variation. The center gives the reference length of 1,022,459 bp. The map displays deposited coordinates, including provisional RNA gene annotations detailed in Table S13; it does not establish a single circular physical molecule.

The mitochondrial reference was annotated with PMGA (no formal version number; accessed on 29 September 2026) against a plant mitochondrial database [24]. Protein-coding features were compared with published *Actinidia* homologs using BLASTN v2.16.0 [25] and inspected in Apollo v1.11.8 [26]. Independent tRNA review used tRNAscan-SE v2.0.13 with -O --max --detail and a eukaryotic sensitivity scan [27]. Both predicted boundaries within 10 bp of an annotation were required for a coordinate match; overlap with only one matching boundary was reported separately as partial support for a compound feature. Organellar scores ≥20 defined the higher-support tier. Anticodon agreement, gene/product/isotype discrepancies, and eukaryotic-model pseudogene flags were retained as separate fields. Covariance-model scores provide computational sequence/structure support, not experimental confirmation of folding or function. For rRNAs, homologous alignments excluding *A. rufa* were used for HMMER v3.3.2 profile searches [28,29]; boundary offsets, strand, aligned coverage, and cross-species sequence comparisons were recorded. These are sequence-profile checks, not direct secondary-structure or motif-by-motif validation. Prediction-only candidates were not added automatically. Table S13 reports the checks and unresolved annotations. The circular coordinate map was drawn with OGDRAW v1.3.1 [30] (Figure 1).

### 2.3. Codon-Usage Analysis

**Figure 2:**
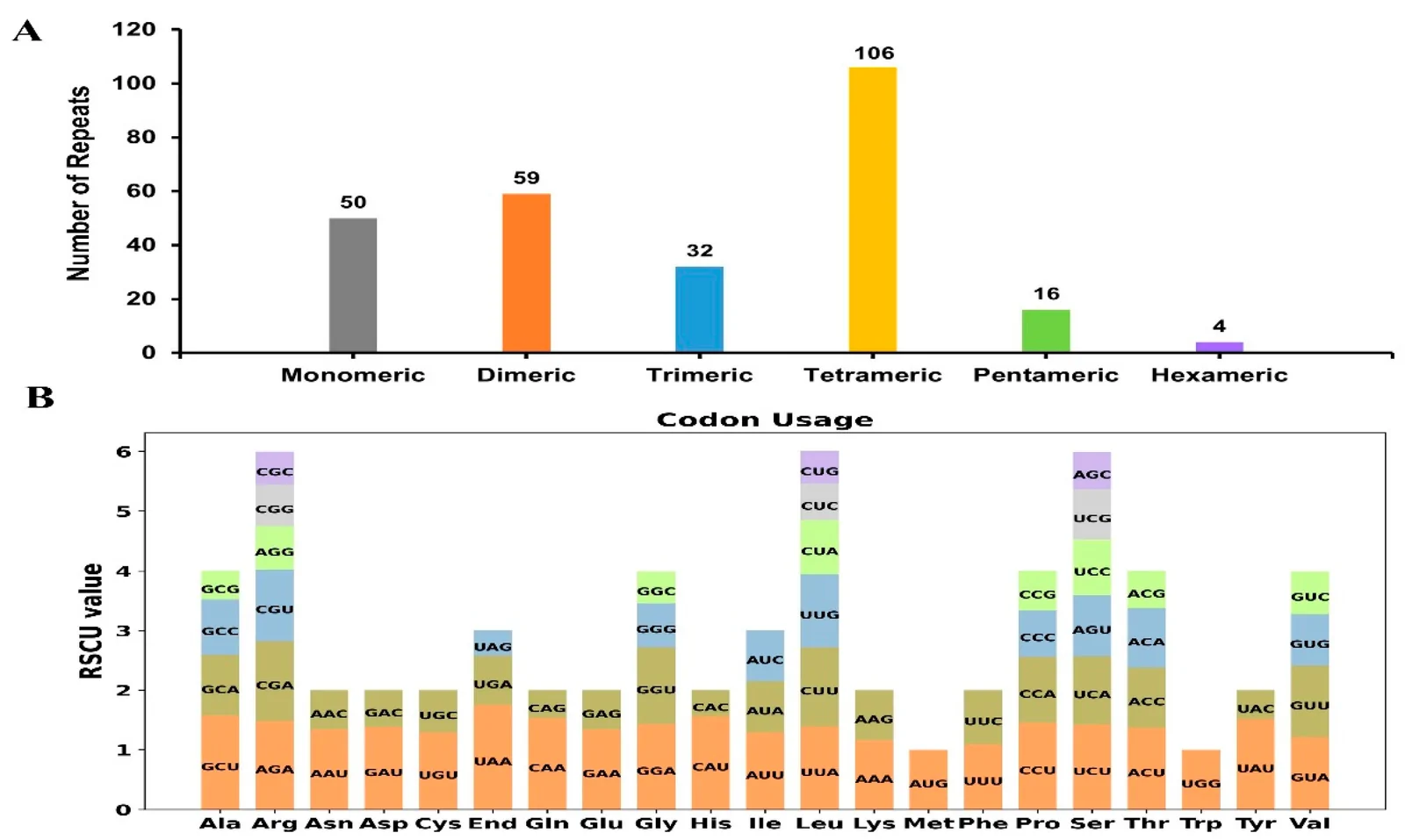
Simple sequence repeats and codon usage in the *A. rufa* mitochondrial reference. (**A**) Numbers of mono- to hexanucleotide SSRs. (**B**) Relative synonymous codon usage (RSCU) for mitochondrial protein-coding genes.

Protein-coding sequences were extracted in PhyloSuite v1.1.16 [31]. Codon counts and relative synonymous codon usage (RSCU) values were calculated in MEGA v12.0.14 [32]. RSCU was defined as the observed frequency of a codon divided by its expected frequency under equal use of synonymous codons; values greater than 1 indicate over-representation. Stop codons were retained in the displayed summary. The codon-usage summary is shown in Figure 2B.

### 2.4. Repeat Detection and Uniform Long-Read Junction Screening

**Figure 3:**
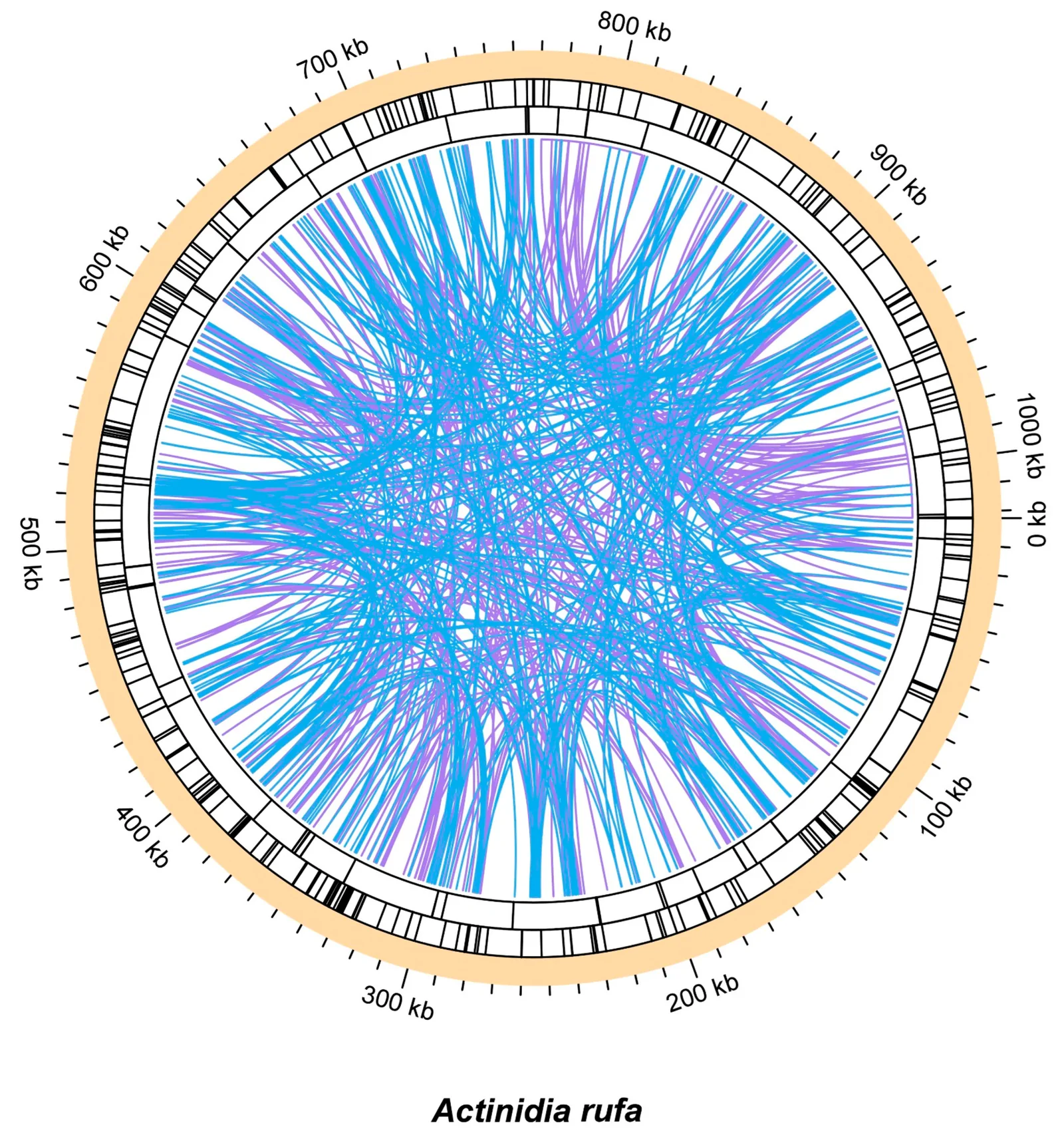
Distribution of dispersed repeats in the *A. rufa* mitochondrial genome. Coloured chords connect paired repeat copies; the dense network illustrates the abundance and genome-wide distribution of forward and palindromic repeats. Repeat discovery used a minimum length of 30 bp.

Simple sequence repeats were detected with MISA v2.1 using minimum repeat-unit thresholds of 10, 5, 4, 3, 3, and 3 for mono- through hexanucleotide motifs [33]. Tandem repeats were identified with Tandem Repeats Finder v4.09 using parameters 2 7 7 80 10 50 500 [34]. Forward and palindromic dispersed repeat pairs ≥30 bp were catalogued with ROUSFinder v2.0 [35]. All 192 pairs ≥50 bp, rather than a subset selected for positive support, were included in the long-read junction screen. This operational cutoff defines the analyzed catalogue; it is not a validated biological threshold for recombination, and no conclusion is drawn about shorter repeats. The SSR counts and dispersed-repeat distribution are shown in Figure 2A and Figure 3, respectively.

For each repeat pair, four synthetic targets represented the two native paths and the two reciprocal alternative paths, each containing the complete repeat plus 2000 bp of sequence on each side. All CRR3429456 reads were aligned using minimap2 map-hifi with secondary alignments retained. A read–path match required ≥99% identity and ≥99% coverage of the synthetic junction target, not of the entire HiFi read. No mapping-quality filter was imposed on these deliberately redundant synthetic targets. Each read was counted once per path; reads supporting both native and alternative classes for the same repeat were excluded. Quantitative evaluability required ≥5 unique reads on each reciprocal native path, and reciprocal alternative support required ≥3 unique reads on each alternative path. These fixed operational rules were applied uniformly and do not constitute experimental validation. For evaluable pairs only, the alternative-path read fraction was calculated as the alternative-class unique-read union divided by the union of native- and alternative-class reads. Two-sided 95% Wilson score intervals describe read-sampling uncertainty within this library [36,37]; they are not confidence intervals for a biological recombination rate. Fractions and intervals for non-evaluable pairs are reported as NA, while their raw counts remain available. Table S5A–E provide counts, exclusions, criteria, coordinates, and nested-repeat relationships.

### 2.5. Mitochondrial–Plastid Homology

**Figure 4:**
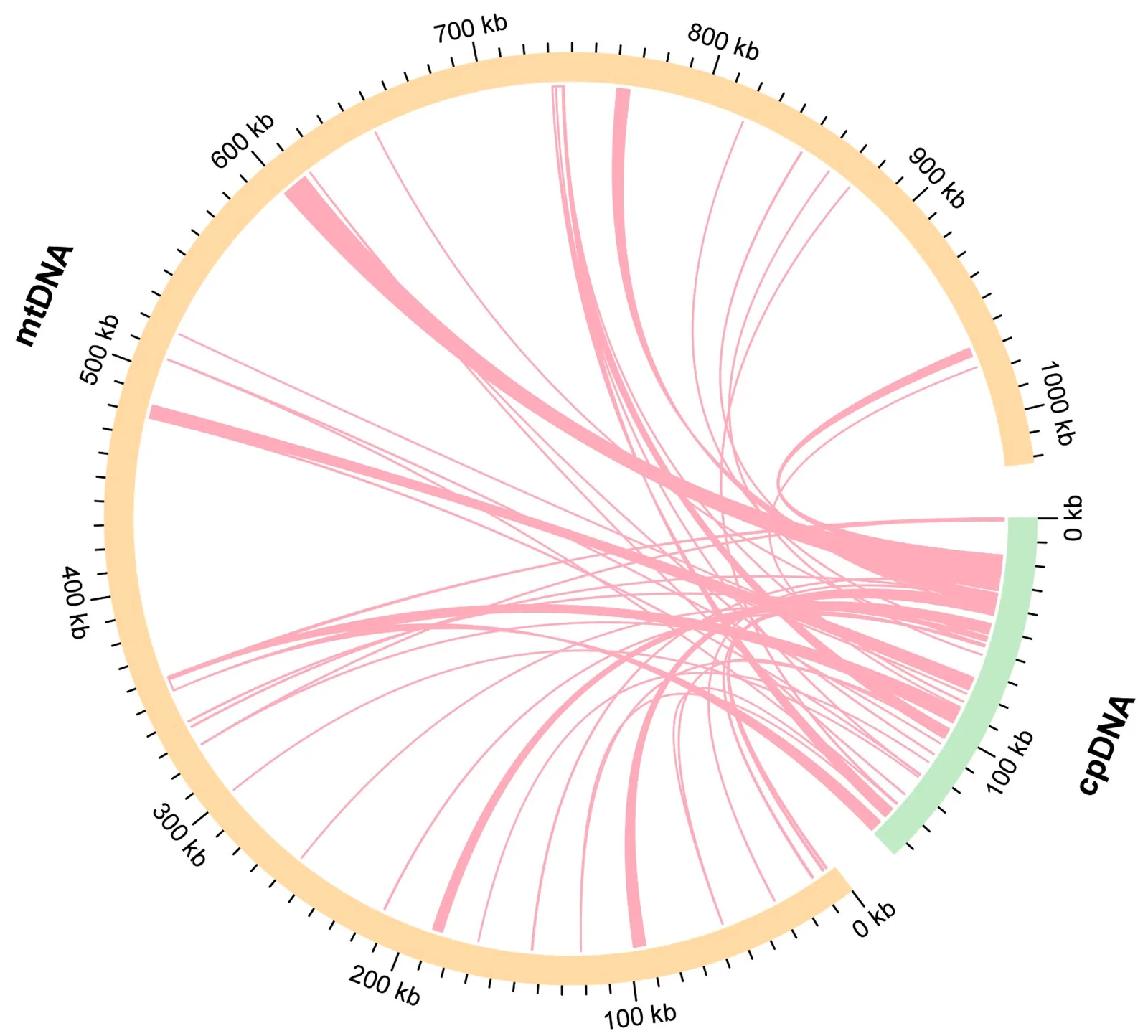
Mitochondrial–plastid homologous fragments in *A. rufa*. Chords link the mitochondrial reference NC_086781.1 and plastome NC_039973.1. The 49 named fragment groups have a representative-HSP length sum of 58,164 bp (5.69%); their nonredundant mitochondrial union is 50,774 bp (4.97%). Overlapping or plastid-repeat-associated hits are not independent transferred regions. Homology alone does not establish transfer direction or function.

The published *A. rufa* plastome NC_039973.1 was used for mitochondrial–plastid comparison [38]. BLASTN matches with E-value ≤ 1 × 10^−5^ were retained and visualized with Circos v0.69-10 [25,39] (Figure 4). Table S6 contains 49 named fragment groups, including additional hits to homologous plastid regions. The representative-HSP length sum is 58,164 bp (5.69% of NC_086781.1), whereas the nonredundant mitochondrial interval union is 50,774 bp (4.97%). The union, not the overlapping HSP sum, was used for reference-side MTPT masking. These matches establish sequence homology, not transfer direction, age, transcription, or function. A matched nuclear assembly was unavailable, so NUMT contributions and the origin of every homologous block could not be resolved.

### 2.6. Phylogenomic Analysis

**Figure 5:**
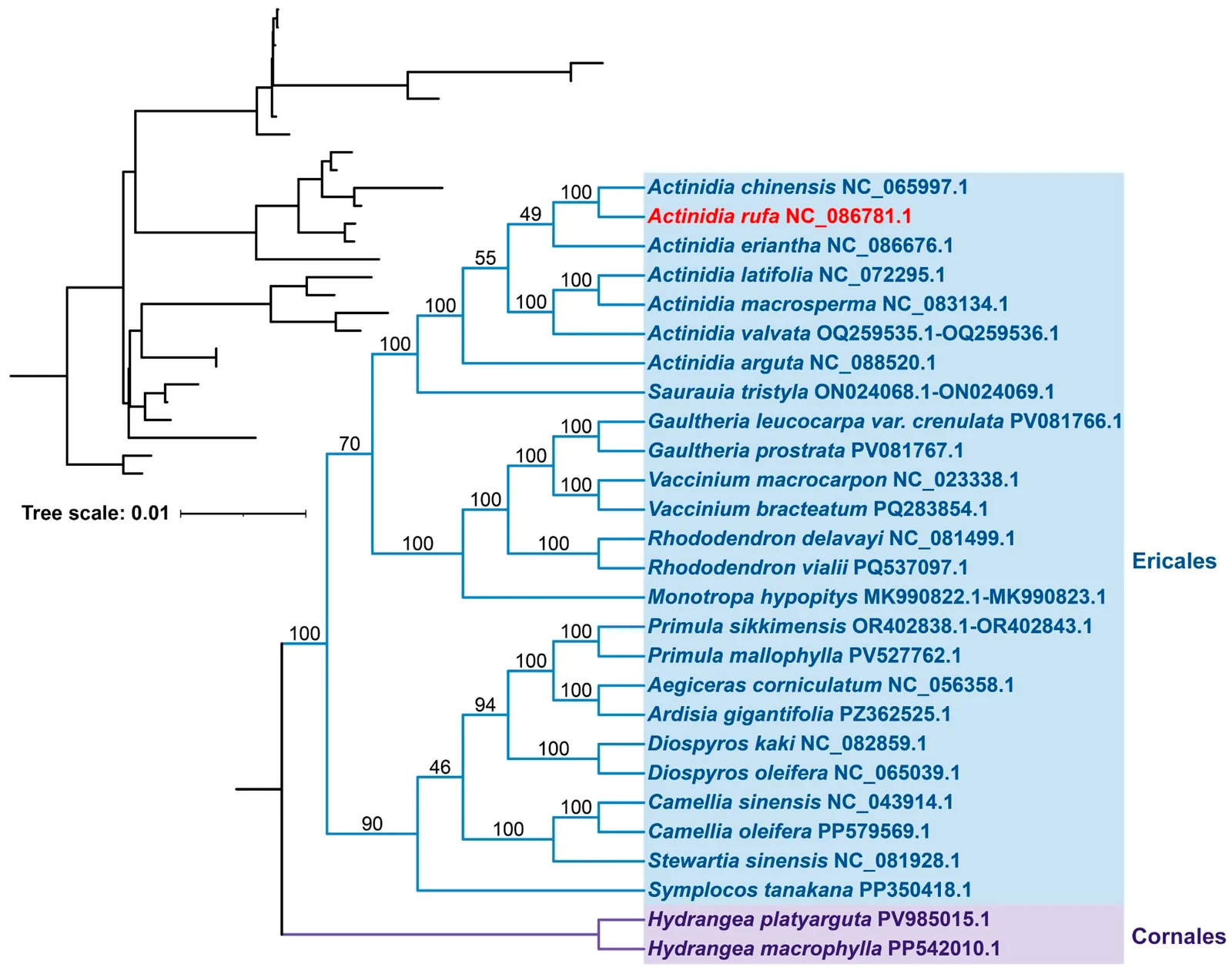
Maximum-likelihood phylogeny inferred from 31 concatenated mitochondrial protein-coding genes for 27 angiosperm taxa. The tree was inferred under GTR+F+R2 with 1000 SH-aLRT and 1000 ultrafast bootstrap replicates. *A. rufa* (NC_086781.1) is highlighted and is sister to *A. chinensis* with maximal support. Two Cornales taxa were used as outgroups.

The broader phylogeny used the 31 mitochondrial protein-coding genes and 27 angiosperm accessions represented in Figure 5. Genes were aligned individually with MAFFT v7.520 [28], concatenated, and analyzed in IQ-TREE v2.2.3 with ModelFinder, 1000 ultrafast-bootstrap replicates, and 1000 SH-aLRT replicates [40]; the tree was visualized in iTOL v7.6 [41]. A separate seven-*Actinidia* analysis evaluated gene-tree concordance. Gene names were normalized, including synonyms, and a gene was retained only if annotated once in every taxon, with minimum/maximum CDS length ratio ≥0.80 and no ambiguous bases. These annotation- and length-based filters reduce obvious missing-data and paralogy problems but do not exhaustively establish orthology. The 31 retained genes were *atp1*, *atp4*, *atp8*, *ccmB*, *ccmC*, *ccmFC*, *ccmFN*, *cob*, *cox1*, *cox2*, *cox3*, *matR*, *mttB*, *nad1*, *nad2*, *nad3*, *nad4*, *nad4L*, *nad5*, *nad6*, *nad7*, *nad9*, *rpl10*, *rpl5*, *rps12*, *rps13*, *rps14*, *rps3*, *rps4*, *sdh3*, and *sdh4*. MAFFT alignments were trimmed using trimAl with gap threshold 0.80 (-gt 0.8; retaining columns with at least 80% non-gap residues) [42], then analyzed using the IQ-TREE support scheme above. A gene was considered informative for the screen if its alignment contained ≥1 parsimony-informative site. We counted trees containing the unrooted *A. rufa* + *A. chinensis* bipartition; this descriptive fraction is not a test of strongly supported biological conflict. Table S12A,B provide annotation-based orthology checks, alignment statistics, and individual topology classifications.

### 2.7. Prediction of RNA-Editing Sites

**Figure 6:**
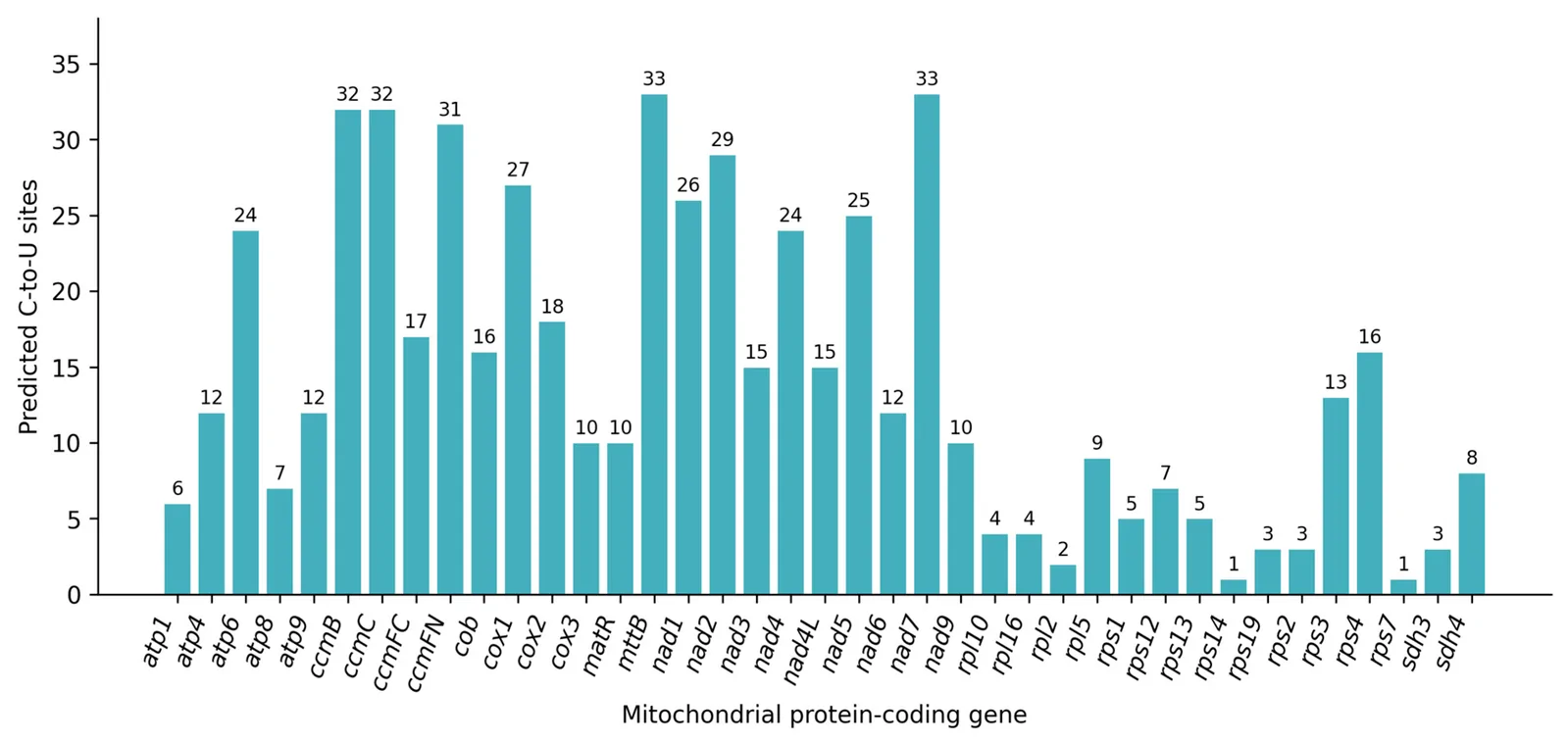
Predicted C-to-U RNA-editing sites in *A. rufa* mitochondrial protein-coding genes. Sites with Deepred-mt probabilities greater than 0.90 are shown. These are computational predictions and have not been validated with RNA sequencing.

Potential C-to-U RNA-editing sites in annotated mitochondrial protein-coding genes were predicted with Deepred-mt (no formal version number; accessed on 29 September 2026) [43]. Only predictions with model probability greater than 0.90 were retained. All 560 predicted sites, including nucleotide and amino-acid positions, codon changes, local sequence context, and probabilities, are reported in Table S7. No matched mitochondrial transcriptome or targeted cDNA data were analyzed; the sites are therefore treated strictly as computational predictions. The predicted-site distribution is summarized in Figure 6.

### 2.8. Comparative Synteny Analysis

NC_086781.1 was compared with *A. chinensis* NC_065997.1, *A. eriantha* NC_086676.1, *A. latifolia* NC_072295.1, *A. macrosperma* NC_083134.1, *A. arguta* NC_088520.1, and *A. valvata* OQ259535.1 and OQ259536.1. The two *A. valvata* components were retained separately, not concatenated. Quantitative comparisons used MUMmer4 nucmer --maxmatch and global one-to-one filtering with delta-filter −1 [44], replacing the earlier qualitative block interpretation. Minimum identity was 90%; minimum alignment length was 500 bp in the primary analysis and 1000 bp in a sensitivity analysis. Fragments were merged only on the same query component and orientation, with reference and query gaps between −100 and 1000 bp and gap discordance ≤1000 bp. Coverage was calculated from the union of filtered aligned intervals rather than merged spans, which can include gaps. One-to-one filtering reduces competing multi-matches but does not prove that all tandem duplications or mapping ambiguities have been eliminated; no dedicated tandem-duplication exclusion was performed.

Candidate discontinuities were defined between consecutive blocks ordered along NC_086781.1: an orientation switch, a same-orientation adjusted query gap <−1000 bp, or reference/query gap discordance >10,000 bp. Query-origin wraps were adjusted using component length; origin-only wraps and the terminal reference adjacency were excluded. Transitions between *A. valvata* components were tabulated separately and excluded from within-component candidate totals. Consecutive reverse-oriented blocks defined candidate reverse intervals, not validated inversions. Dispersed-repeat and MTPT overlap was recorded within ±1 kb of candidate boundaries and across reverse intervals; overlap counts are descriptive, not enrichment tests. Independent sensitivity analyses masked only the *A. rufa* reference using the 111,377 bp dispersed-repeat union and the 50,774 bp MTPT union before realignment. To evaluate the hypothesized CDS versus non-CDS coverage difference, CDS coverage used the union of all annotated CDS parts (32,700 bp); non-CDS comprised all other reference bases (989,759 bp), including RNA genes and introns as well as intergenic sequence. Positional enrichment was assessed with a circular-shift test preserving the observed alignment mask, without treating bases as independent observations. The Benjamini–Hochberg adjustment was applied to the six unmasked primary-comparison *p* values [45]; masked comparisons were retained as sensitivity analyses. Assemblies were not reassembled uniformly, and comparisons cannot independently validate their physical structures. Tables S8–S13 include the analysis criteria and supporting results.

## 3. Results

### 3.1. Reference Gene Content and Annotation Support

To establish the reference features underlying the read-based and comparative analyses, we examined the gene content of NC_086781.1, a 1,022,459 bp circular reference representation with a GC content of 45.65% (Figure 1). Its annotation contains 39 protein-coding loci, 37 tRNA loci representing 23 named tRNA types, and three rRNA loci (Table 1 and Table S1). The circular coordinate map is a convenient reference display and is not interpreted as proof that one circular molecule is the sole physical form.

The protein-coding complement includes 24 core and 15 variable mitochondrial loci. Independent RNA gene checks supported most annotated coordinates but left specific loci unresolved. The tRNA screen returned 40 organellar-model predictions: 35 annotations matched at both boundaries, one compound annotation had partial boundary support, and one annotated locus was not detected. Of the 36 annotation-associated predictions, 33 scored ≥20 and three scored <20; the latter include the partial match. Four prediction-only candidates were not added. The eukaryotic sensitivity scan yielded 34 predictions, 33 annotation matches, and two provisional pseudogene flags. Gene/product/isotype discrepancies at EI095_mgt14, EI095_mgt26, and EI095_mgt37 were retained as unresolved annotation issues. All three rRNAs had sequence-profile support: *rrn5* and *rrn18* boundaries matched exactly, whereas *rrn26* differed by 5 bp at the lower genomic boundary and 1 bp at the upper boundary, with 99.84% annotated-length coverage (Table S13). These checks support many reference coordinates but do not establish expression, processing, secondary structure in vivo, or function.

### 3.2. Codon Bias and the Repeat Set for Junction Screening

The 39 PCGs exhibited a preference for codons ending in U or A (Figure 2B; Table S2), providing a compositional description of the annotated coding sequences. UAA had the highest stop-codon RSCU value (1.75), and GCU was an over-represented sense codon (1.57). The complete codon-usage profile is retained in Table S2; this analysis was not used to infer translational selection.

To define the candidate set for evaluating repeat-associated junctions, we catalogued 267 SSRs, 57 tandem repeats, and 736 dispersed repeat pairs of at least 30 bp (Figure 2 and Figure 3; Table 2, Tables S3 and S4). The dispersed-repeat catalogue comprised 426 forward and 310 palindromic pairs. The 192 pairs of at least 50 bp were advanced to uniform long-read junction screening; repeat length alone was not interpreted as evidence of recombination.

### 3.3. Partial Reference Coverage and Limited Alternative Junction Support

HiFi mapping supported a part, but not all, of the mitochondrial reference under the specified filters. Stringent competitive mapping of CRR3429456 against NC_086781.1 and NC_039973.1 gave a mean mitochondrial-reference depth of 40.35× and a median depth of 48×. Breadth was 73.9% at ≥1×, 72.4% at ≥10×, 70.2% at ≥20×, 45.4% at ≥50×, and 1.4% at ≥100× (Figure S2); the read-length distribution is shown in Figure S1. No uniquely spanning terminal-to-start read was recovered with 1, 2, or 5 kb anchors. These results delimit the read support for reference coordinates. Uncovered coordinates cannot be assigned uniquely to sequence absence, divergence, repeats, or filtering effects, and the observations do not establish the predominant physical molecule.

The uniform screen did not identify any repeat pair meeting the operational rule for reciprocal alternative-junction support. All 192 repeat pairs ≥50 bp were screened under identical rules (Figure S3; Table S5A–E). Forty-one met the reciprocal-native-path evaluability rule: 39 had no alternative-path read, while R43 (99 bp, palindromic) and R47 (96 bp, palindromic) each had one read on one alternative path and none on the reciprocal path. Their descriptive alternative-path read fractions were 1/81 (1.23%; Wilson 95% CI, 0.22–6.67%) and 1/85 (1.18%; 0.21–6.37%), respectively (Table 3). Neither met the reciprocal-alternative-support rule. The other 151 pairs, including R1 and R5, were not evaluable; their fractions and intervals are therefore NA. R21, R92, R119, and R135 had alternative-only matches without adequate reciprocal native support; R135 is nested within R92. Thus, the available reads do not establish reciprocal alternative configurations under the tested criteria; the negative screen is not evidence that recombination is absent.

### 3.4. Plastid Homology Defines a Limited Reference Fraction

Plastid-homologous intervals occupied 50,774 bp (4.97%) of NC_086781.1 after overlapping mitochondrial intervals were merged (Figure 4; Table S6). This nonredundant union defined the reference-side MTPT mask used to assess its contribution to interspecific alignment coverage (Section 3.7). The 49 named fragment groups had a larger summed representative-HSP length of 58,164 bp (5.69%). The distinction prevents overlapping HSPs from being interpreted as unique genomic coverage. These similarity matches do not determine transfer direction or function.

### 3.5. Concatenated Placement with Limited Single Gene Concordance

To assess whether individual genes consistently recovered the concatenated placement, we compared the focal *A. rufa*–*A. chinensis* relationship across analyses. The broader concatenated analysis placed *A. rufa* sister to *A. chinensis* with maximal displayed branch support (Figure 5). The separate seven-*Actinidia* alignment contained 28,578 positions, 148 variable sites (0.52%), and 22 parsimony-informative sites (0.08%). Fifteen of the 31 genes had no parsimony-informative site. Among 16 informative gene trees, eight contained the *A. rufa* + *A. chinensis* bipartition and eight did not; the latter included one tree without an *A. rufa* two-taxon sister split. Ten genes had no variable sites and 28 had at most five. Thus, the 50% focal-split concordance is descriptive, and alternative resolutions cannot automatically be interpreted as strongly supported biological conflict (Figure S7; Table S12A,B). The topology represents mitochondrial sequence history rather than a comprehensive species history.

### 3.6. Predicted RNA Editing as Supporting Reference Characterization

As a supporting resource for future transcript-based evaluation, Deepred-mt returned 560 C-to-U editing predictions with model probability greater than 0.90 (Figure 6; Table S7). The largest predicted counts occurred in *mttB* and *nad7* (33 each), followed by *ccmB* and *ccmC* (32 each) and *ccmFN* (31). These values are model outputs; no site, editing fraction, transcript, tissue difference, or functional effect was experimentally validated.

### 3.7. Higher CDS Coverage Despite Variable Alignment Block Organization

Substantial interspecific sequence coverage was distributed among multiple forward and reverse blocks. At the 500 bp threshold, filtered alignments covered 69.94–88.18% of NC_086781.1 and 70.63–97.44% of query sequence (Figure S4; Table S9A,B). Across six species comparisons there were 262 merged blocks: 156 forward and 106 reverse, with 32–61 blocks per comparison. *A. chinensis* gave 88.18% reference coverage in 45 blocks (25 forward, 20 reverse). The two *A. valvata* components contributed 44 blocks (23 forward, 21 reverse) and 17 blocks (6 forward, 11 reverse), respectively. The 1000 bp sensitivity results are reported separately. The candidate catalogue contains 174 within-component coordinate discontinuities (Table S10A), 66 reverse-oriented intervals (Table S10B), and 22 *A. valvata* component transitions (Table S10C). Component transitions are bookkeeping changes in the query component, not inferred physical junctions or rearrangement breakpoints.

The CDS versus non-CDS comparison directly addressed the study hypothesis. Unmasked CDS coverage was 99.90–100%, compared with 68.94–87.79% for non-CDS sequence, a difference of 12.21–31.06 percentage points. Circular-shift *p* values ranged from 0.000566 to 0.008745; the Benjamini–Hochberg-adjusted values across the six primary comparisons ranged from 0.001674 to 0.008745 (Figure S5B; Table S11B). Thus, CDS coordinates had higher alignment coverage in all six comparisons, while the block counts and orientations documented variable reference organization. Higher CDS alignment coverage does not demonstrate conserved gene order, equal nucleotide identity, or a particular mutational mechanism.

Separate masking analyses assessed whether overall sequence coverage depended mainly on annotated repeats or MTPTs. Reference-side repeat masking retained 94.39–98.29% of baseline aligned reference sequence, and MTPT masking retained 93.60–99.39% (Figure S5A; Table S11A). Most baseline alignment coverage therefore remained after either mask. Dispersed repeats overlapped a ±1 kb window at one or both boundaries of 127/174 within-component candidates (72.99%); MTPTs overlapped 34/174 (19.54%). Repeat and MTPT overlaps occurred in 52/66 (78.79%) and 31/66 (46.97%) reverse intervals, respectively. These overlap proportions are descriptive associations, not evidence of causation or enrichment.

## 4. Discussion

### 4.1. A Large Reference Without Coding-Sequence Expansion

The higher CDS than non-CDS alignment coverage supports the stated sequence-retention hypothesis within the six reference comparisons. The 1,022,459 bp *A. rufa* reference is large within *Actinidia* but falls within the broad size variation of angiosperm mitogenomes. Large genomes in *Cucurbita*, *Cucumis*, *Amborella*, and *Silene* illustrate expansion through intergenic accumulation, foreign-sequence acquisition, or multipartite organization rather than proportional enlargement of the conserved respiratory gene set [46,47,48,49,50]. Accordingly, 39 annotated protein-coding loci do not scale directly with the total reference length. Plant mitochondrial coding sequences can evolve slowly while genome organization changes rapidly [5,51,52]. Our six comparisons show near-complete CDS alignment coverage and lower non-CDS coverage, with significant circular-shift results after adjustment across the six primary comparisons. This is a coordinate-coverage contrast; it neither measures the stability of coding-gene order nor establishes that sequence evolution and structural evolution are statistically independent [5,51,52].

U- and A-ending codon preferences are descriptive and cannot distinguish mutational bias from translational selection. Likewise, 37 annotated tRNA loci with 23 deposited gene names do not establish an autonomous mitochondrial translation system, because plant mitochondria can import nuclear-encoded tRNAs [53]. Independent model-based annotation review supports many RNA gene coordinates but leaves low-score, partial-boundary, identity-discrepancy, and provisional pseudogene cases unresolved. Computational model support is not evidence for transcription, processing, correct folding in vivo, or aminoacylation [27,53].

### 4.2. Repeat-Junction Evidence Is Sparse and Heterogeneous

For the objective of assessing repeat-associated junctions, the uniform all-pair screen establishes the limits of the current read evidence. Of 192 pairs, 41 met the native-path evaluability rule and none met the reciprocal-alternative-support rule. R43 and R47 each had a single read on only one alternative path. These observations identify low-count candidate junctions, not confirmed alternative configurations or ongoing recombination. Repeat abundance, perfect identity, and circular coordinate representation are insufficient by themselves to demonstrate activity [10,11,12,13,35,52].

Alternative mitochondrial forms have been investigated in diverse plants, but their measured abundances depend on repeat context, genotype, tissue, sequencing design, and counting rules [54,55,56,57,58,59,60,61,62,63]. Those studies provide context, not frequencies transferable to *A. rufa*. R5 was not evaluable under the uniform rule, so its earlier frequency estimate is not retained. Alternative-only matches for R21, R92, R119, and R135 likewise lack a usable native-path denominator; nesting of R135 within R92 further limits independence. The Wilson intervals for R43 and R47 quantify only within-library read sampling and cannot establish a biological configuration frequency. Independent junction-specific testing or additional long-read libraries would be needed to resolve such low-count signals [54,55,60,61,62,63].

The 43,947 bp perfect forward repeat R1 was not quantitatively evaluable. Its length and redundancy complicate unique junction assignment even though some reads are longer than the repeat-plus-flank target. Non-evaluability is not evidence of inactivity. Ultra-long reads, targeted enrichment, or junction-specific assays remain appropriate ways to assess its possible molecular arrangements [10,12,64,65].

### 4.3. A Circular Coordinate Reference Does Not Define the Physical Molecule

NC_086781.1 provides a circular coordinate system, whereas plant mitochondrial DNA may include linear, branched, circular, and subgenomic forms [12,55,64,65]. Stringent mapping covers only part of the reference and no unique terminal-to-start read was found under the tested criteria. These observations do not prove either a circular or a noncircular architecture, nor do they establish completeness of the deposited physical arrangement. The reference remains useful for annotation and coordinate-based comparisons, provided that its physical interpretation is kept separate from its sequence representation [64,65].

Nuclear-encoded recombination surveillance proteins such as RECA and MSH1 provide plausible mechanisms for maintaining plant mitochondrial genomes, and experimental disruption of these pathways can alter ectopic exchange and substoichiometric forms [66,67,68,69]. No nuclear-genotype, protein-function, or perturbation experiment was performed here. Nuclear control is therefore retained only as a future mechanistic hypothesis and not as an explanation established for *A. rufa* [66,67,68,69].

### 4.4. Quantified Similarity Patterns and Their Limits

The comparative objective was to determine how much homologous sequence was retained and how it was organized among references. High retention of baseline alignment coverage after either repeat or MTPT masking indicates that the overall coverage pattern is not accounted for mainly by either annotated feature class. These separate masks do not establish that the CDS versus non-CDS contrast is independent of every repeat or transferred segment. The 174 within-component discontinuities and 66 reverse intervals are hypotheses for further assessment, not confirmed inversions or translocations. Public assemblies differ in sequencing, assembly, repeat resolution, and representation; standardized alignment cannot remove these source-level differences [19,20,21,22,51,52]. Large size, repeats, transferred sequence, and disrupted collinearity also occur in *Vitis*, *Cucumis*, *Silene*, and *Brassica* [47,49,50,70,71]. These are contextual examples, not a matched cross-genus benchmark, and our results do not show that *Actinidia* has an unusually high rearrangement rate.

The coexistence of high CDS coordinate coverage and variable block organization supports the operational hypothesis, not an independence or “decoupling” of gene-sequence and structural evolution [5,6,51]. The phylogenetic objective has a more qualified outcome: the concatenated placement is not consistently recovered by the informative individual genes, but the data cannot distinguish weak resolution from strongly supported alternative histories. The seven-*Actinidia* alignment contains few variable and parsimony-informative sites, so limited phylogenetic information can contribute to alternative single-gene resolutions. The 8/16 focal-split fraction should not be treated as a strongly supported conflict statistic. Although the concatenated *A. rufa*–*A. chinensis* relationship is consistent with the cited nuclear study, the mitochondrial analysis is a complementary organellar perspective and does not establish the complete species history [5,6,18].

### 4.5. Organelle Homology and Computational RNA-Editing Predictions

The mitochondrial–plastid comparison demonstrates why overlapping HSP totals and nonredundant coverage must be distinguished. The representative-HSP sum is 5.69%, whereas the mitochondrial union is 4.97%. Homology alone does not determine the direction or timing of transfer, transcription, or function [14,15,72,73]. Reference-side MTPT masking retained 93.60–99.39% of baseline aligned reference sequence, indicating that plastid-homologous intervals do not account for most observed alignment coverage. However, this does not establish true mitochondrial ancestry for every retained block. Without a matched nuclear assembly, NUMT-related contributions and transfer direction remain unresolved; indiscriminately deleting every nuclear-homologous region would also be unjustified [14,72,73].

All 560 RNA-editing sites are computational predictions. Their gene distribution is broadly compatible with editing reported in other angiosperms, but cross-species plausibility is not validation in *A. rufa* [16,43,74,75,76,77]. Table S7 provides the complete prediction list and model probabilities. Matched mitochondrial RNA sequencing or targeted cDNA sequencing is required to confirm individual sites, quantify editing proportions, and test tissue or waterlogging responses [74,75,76,77].

### 4.6. Study Limitations and Priorities for Validation

Several limitations delimit the conclusions. One mitochondrial reference and one HiFi library do not permit population-level inference. Stringent mapping covered 73.9% of NC_086781.1 at ≥1× and recovered no unique terminal-to-start read at the tested anchors; the reference’s complete physical architecture is therefore not established. Only 41 of 192 repeats were quantitatively evaluable, R43 and R47 had one-sided single-read support, and R1 and R5 were not evaluable. No junction-specific PCR, qPCR, droplet digital PCR, independent library, or tissue replicate was analyzed. Within-library confidence intervals cannot substitute for such replication, particularly where genome organization and molecule abundance can depend on DNA-repair mechanisms [78].

The comparative results depend on heterogeneous public assemblies; candidate discontinuities and reverse intervals are not verified evolutionary events. Repeat/MTPT masking is reference-sided, NUMTs remain unresolved, and the CDS contrast concerns coordinate coverage rather than conserved gene order. The gene-tree screen has limited phylogenetic information and cannot establish a species tree [79]. RNA editing lacks transcriptomic validation, and RNA gene model support does not establish functional transcripts [80]. No waterlogging treatment or mitochondrial functional assay was performed. Priorities are independent junction testing, targeted evaluation of R1, population and tissue replication, matched transcriptomics, and standardized source-read-supported comparative assemblies. Supported observations include reference features, filtered read support, repeat counts, quantitative alignment metrics, model predictions, and descriptive topology differences. Physical architecture, ongoing recombination, nuclear control, structural adaptation, and a mitochondrial contribution to waterlogging tolerance remain untested [78,79,80].

## 5. Conclusions

The six *Actinidia* comparisons support the hypothesis that CDS coordinates retain higher interspecific alignment coverage than non-CDS coordinates despite variable alignment block organization. CDS coverage was 99.90–100%, compared with 68.94–87.79% for non-CDS sequence, and the contrast remained significant after adjustment across the six primary comparisons. Most baseline aligned reference sequence was retained after separate repeat and MTPT masking. For the read-support objective, NC_086781.1 provides an annotated coordinate resource, but filtered HiFi coverage and the repeat-junction screen do not establish its complete physical architecture or ongoing recombination: 41 of 192 repeat pairs were evaluable, none met the reciprocal-alternative-support rule, and R1 and R5 remained non-evaluable. For the phylogenetic objective, eight of 16 informative gene trees contained the *A. rufa*–*A. chinensis* split, with limited information constraining interpretation. RNA-editing predictions and unresolved RNA gene annotations remain resources for validation. These findings define supported sequence-comparison patterns and the limits of structural inference; they do not demonstrate a mitochondrial contribution to waterlogging tolerance.

## Figures and Tables

**Table 1:** Principal features of the *Actinidia rufa* mitochondrial genome.

Feature	Value
GenBank/RefSeq accession	NC_086781.1
Reference representation	Circular coordinate sequence
Genome size	1,022,459 bp
GC content	45.65%
Protein-coding loci	39 (24 core; 15 variable)
tRNA annotation	37 loci; 23 deposited gene names
rRNA annotation	3 loci (*rrn5*, *rrn18*, *rrn26*)
Predicted C-to-U RNA-editing sites	560 (probability >0.90; computational only)

Note: tRNA counts distinguish 23 deposited gene names from 37 annotated loci, not 23 experimentally verified functional types. Table S13 retains boundary, identity, low-score, and provisional pseudogene uncertainties.

**Table 2:** Repetitive elements in the *A. rufa* mitochondrial genome.

Repeat Class	Criterion	Count	Key Observation
SSRs	MISA 10/5/4/3/3/3	267	Tetranucleotides: 106 (39.70%)
Tandem repeats	TRF; 2–86 bp	57	Identity at least 64%
Dispersed repeats	Length at least 30 bp	736 pairs	426 forward; 310 palindromic
Junction-screen set	Length at least 50 bp	192 pairs	41 evaluable under native-path rule
Longest dispersed repeat	R1; 43,947 bp	1 pair	Not quantitatively evaluable

**Table 3:** Uniform long-read junction-screening summary for the 192 dispersed repeat pairs of at least 50 bp.

Screen Category	Length/Type	Native Paths	Alternative Paths	Alt./Total Reads	Wilson 95% CI	Interpretation
R43	99 bp/Pal.	39/41	1/0	1/81 (1.23%)	0.22–6.67%	One-sided; insufficient
R47	96 bp/Pal.	52/32	1/0	1/85 (1.18%)	0.21–6.37%	One-sided; insufficient
Other evaluable	39 pairs	At least 5/5	0/0	0	See Table S5A; pair-specific	No alternative detected
Not evaluable	151 pairs	Below 5 on at least one path	Raw counts in Table S5A	NA	NA	Includes R1 and R5
All screened	At least 50 bp	192 pairs	Uniform 2 kb-flank screen	41 evaluable	Pair-specific; see Table S5A	No reciprocal alternative support

Note: Evaluability required ≥5 unique reads on each native reciprocal path; reciprocal alternative support required ≥3 on each alternative reciprocal path. Fractions and Wilson intervals are reported only for evaluable pairs and describe read sampling within one library, not biological configuration frequencies.

## Data Availability

The *A. rufa* mitochondrial reference is available in NCBI GenBank/RefSeq under NC_086781.1. PacBio Revio HiFi reads analyzed here are available in the Genome Sequence Archive under BioProject PRJCA071801, accession CRA048345, run CRR3429456. The plastome used for MTPT analysis is NC_039973.1. Derived read-support, repeat-junction, synteny, masking, feature-coverage, gene-tree, RNA-editing, and annotation-review results are provided in the Supplementary Tables and Figures.

## Supplementary Materials

The following supporting information can be downloaded at: https://www.mdpi.com/article/10.3390/genes17101209/s1, Supporting information comprises Figures S1–S7: HiFi read lengths, stringent reference coverage, uniform junction screening, quantitative synteny, masking/feature-class coverage, structural dot plots, and gene-tree concordance. Tables S1–S4 contain deposited annotations, codon usage, SSR/tandem repeats, and dispersed repeats. Table S5A–E contain junction counts, candidate rows, and analysis metadata; Table S6, mitochondrial–plastid homologous fragments; Table S7, all predicted RNA-editing sites; Table S8, comparative accessions and source-information scope [19,20,21,22]; Table S9A,B, quantitative alignments and merged blocks; Table S10A–C, within-component candidate discontinuities, reverse intervals, and component transitions; Table S11A,B, masking sensitivity and CDS versus non-CDS coverage; Table S12A,B, gene-tree/alignments and annotation-based orthology checks; Table S13, RNA gene annotation review. An analysis-criteria sheet documents the decision rules.
