# Supplementary material for: Characterization and Comparative Analysis of the Actinidia rufa Mitochondrial Genome
Source: Genes (Basel). 2026 Sep 29;17(10):1209. doi: 10.3390/genes17101209 (PMC13704310; doi:10.3390/genes17101209)
Supplement: Supplementary file 1 [file genes-17-01209-s001.zip › Supplementary_Figures_S1-S7/Actinidia_rufa_Supplementary_Figures_and_Legends_v3.pdf]

Supplementary Figures S1–S7

Characterization and Comparative Analysis of the *Actinidia rufa* Mitochondrial Genome

The seven figures and legends are synchronized with the v3 manuscript and Supplementary Tables S1–S13. Source read identifier: CRR3429456. The originally main-text repeat-screen and quantitative-synteny panels are supplied only as Figures S3 and S4.

Figure S1

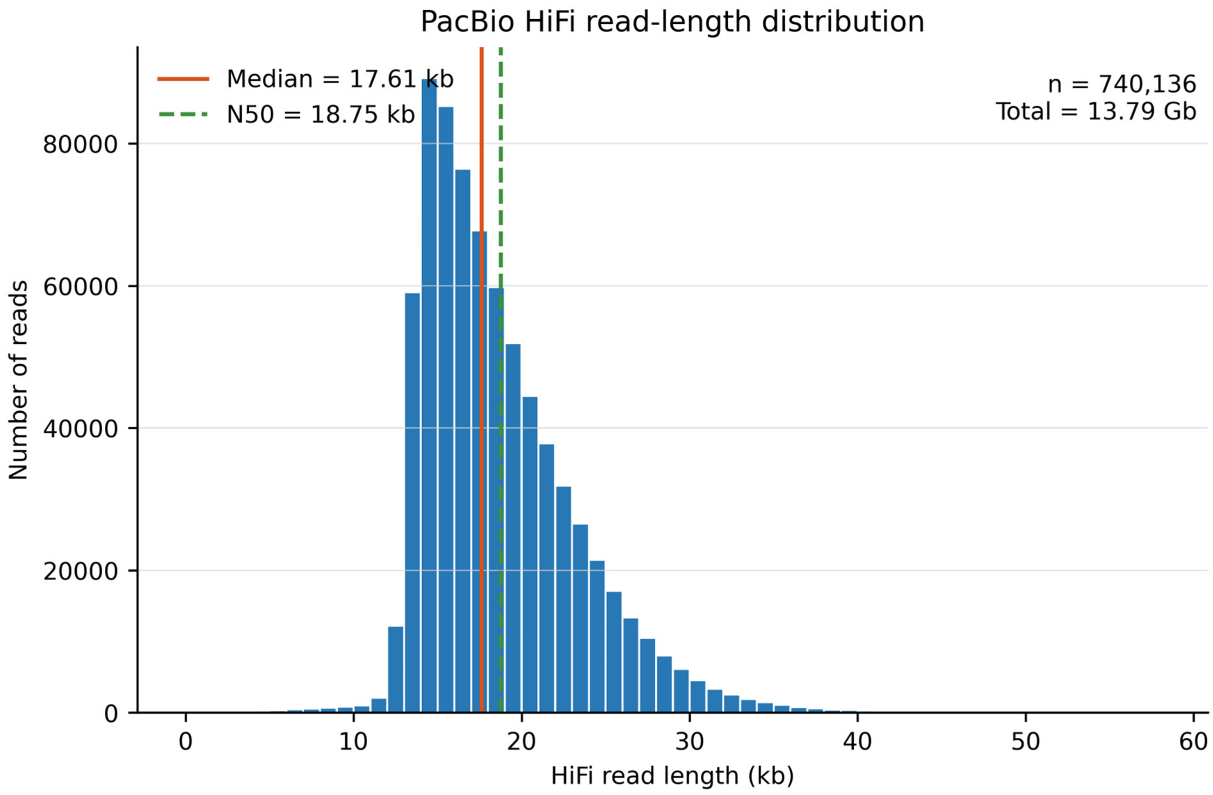

Figure S1. PacBio HiFi read-length distribution for CRR3429456. All 740,136 reads total 13.79 Gb. Median length is 17,615 bp, read N50 is 18,748 bp, and maximum length is 57,759 bp. The histogram describes the whole library, not only mitochondrial-assigned reads.

Related response: Reviewer 1, comments 1–2; Section 3.3; Table S5C.

Figure S2

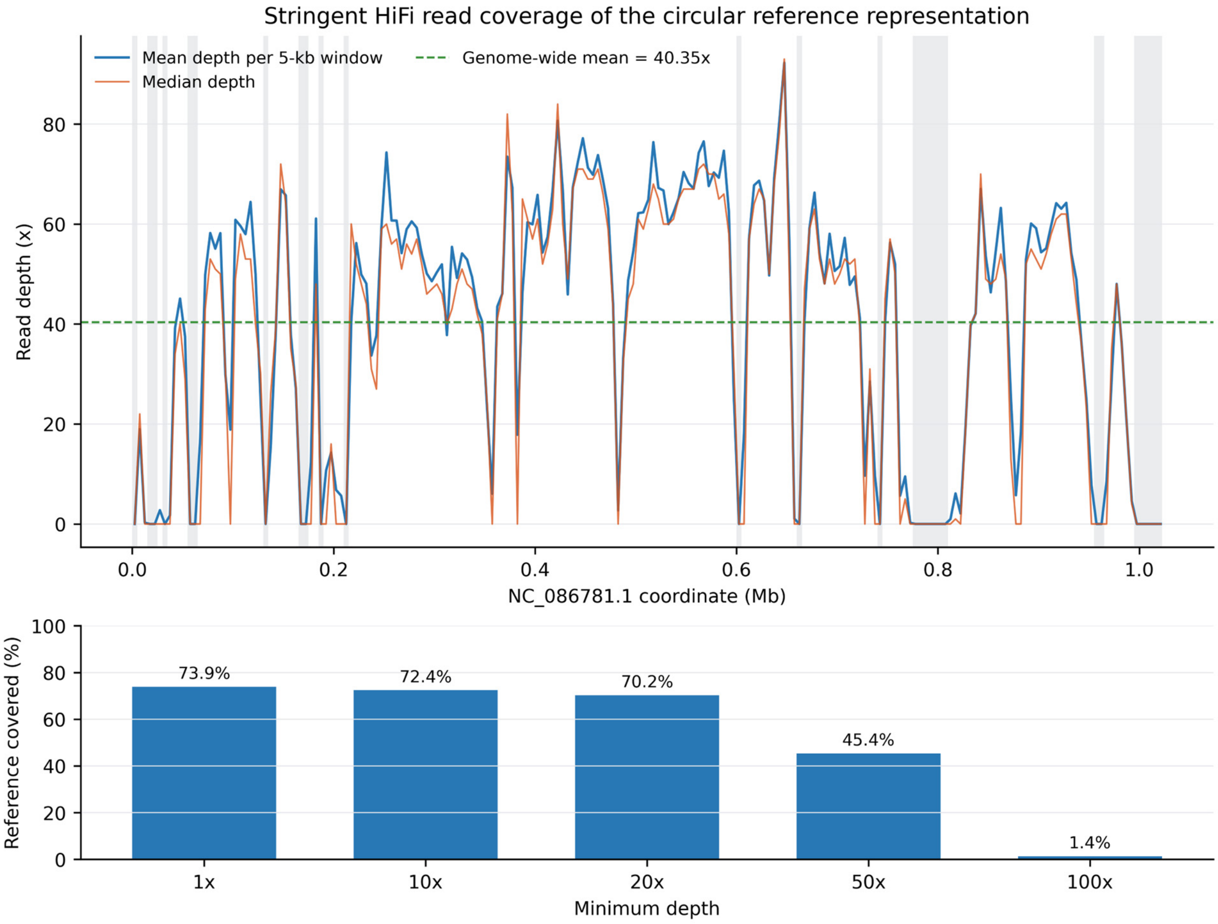

Figure S2. Stringent competitive HiFi coverage of NC_086781.1. CRR3429456 reads were mapped against mitochondrial and plastid references; primary mitochondrial alignments with MAPQ ≥20 and bases with quality ≥10 were retained. Upper panel: mean/median depth in 5-kb windows; the horizontal line marks mean depth 40.35×. Shading identifies intervals with low filtered coverage, without attributing a unique cause. Lower panel: reference breadth at ≥1×, ≥10×, ≥20×, ≥50×, and ≥100× is 73.9%, 72.4%, 70.2%, 45.4%, and 1.4%, respectively. Filtered coverage does not establish complete sequence or physical-architecture validation.

Related response: Reviewer 1, comments 1–2; Sections 2.2 and 3.3; Table S5C.

Figure S3

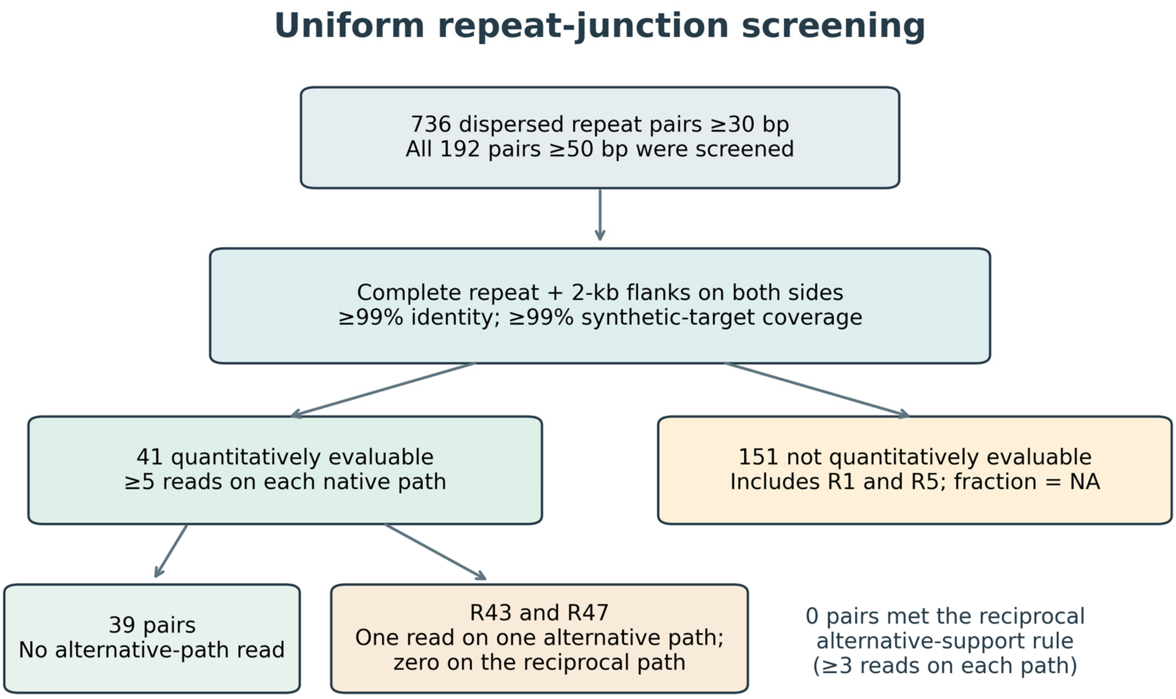

Figure S3. Uniform repeat-junction screening using CRR3429456. Of 736 dispersed repeat pairs ≥30 bp, all 192 pairs ≥50 bp were tested with complete repeats and 2-kb flanks on each side. Matches required ≥99% identity and ≥99% synthetic-target coverage; ambiguous native/alternative read assignments were excluded. Forty-one pairs met the ≥5-reads-per-native-path rule: 39 had no alternative-path support and R43/R47 each had one read on one alternative path only. The other 151 pairs, including R1 and R5, were not quantitatively evaluable and have NA fractions/intervals. No pair met the ≥3-reads-per-reciprocal-alternative-path rule. This is an operational junction screen, not confirmation of ongoing recombination or its absence.

Related response: Reviewer 1, comments 3–7; Reviewer 2, comment 3; Tables S5A–S5E.

Figure S4

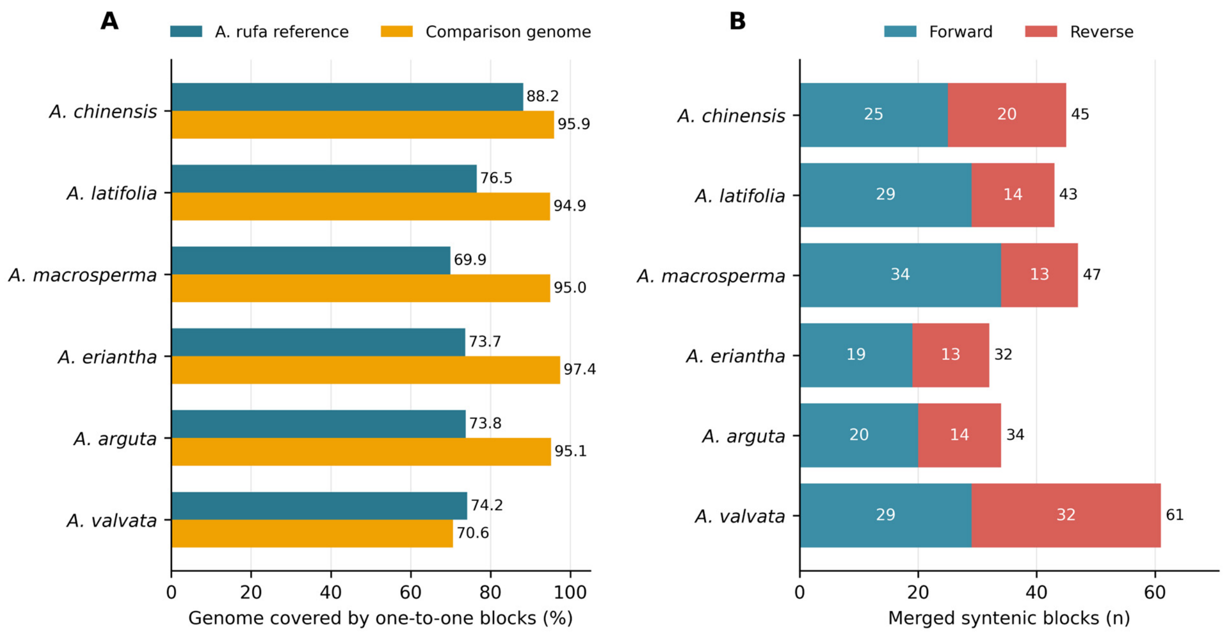

Figure S4. Quantitative one-to-one mitochondrial alignments between *A. rufa* and six congeners. (A) Reference and query coverage calculated from unions of filtered aligned intervals. (B) Merged forward/reverse block counts. The pipeline used nucmer --maxmatch, delta-filter -1, ≥90% identity, and minimum length 500 bp. Merging required the same component/orientation, gaps between −100 and 1,000 bp, and gap discordance ≤1,000 bp. *A. valvata* components remain separate, with combined counts displayed only as a species-level summary. Orientation differences are not automatically evolutionary inversions.

Related response: Reviewer 1, comment 13; Reviewer 2, comments 1–2 and 7; Tables S8–S9.

Figure S5

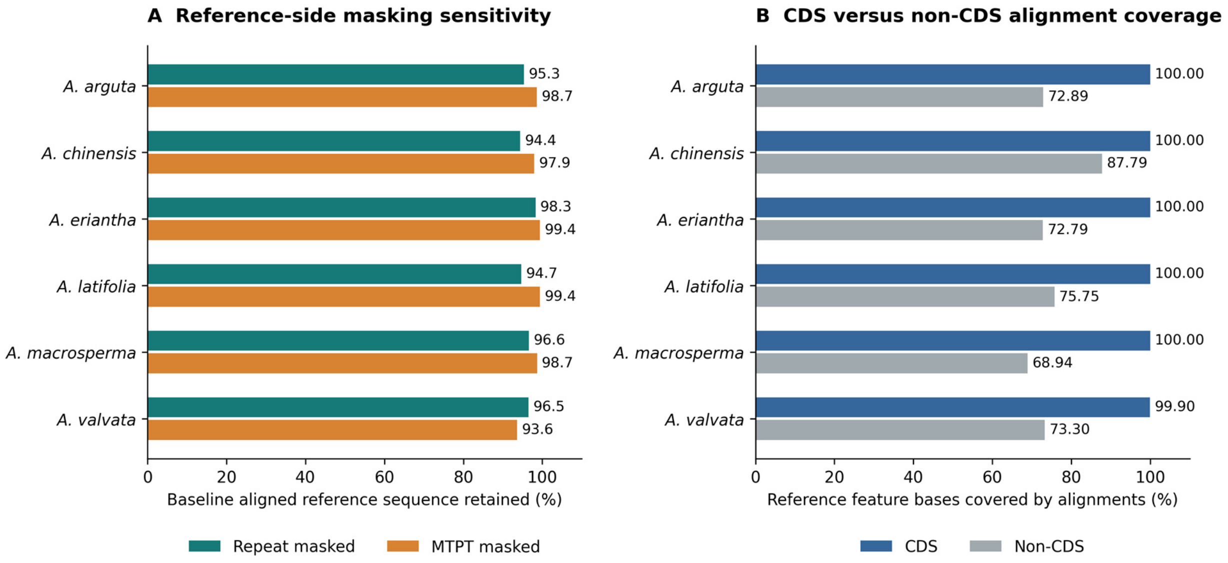

Figure S5. Reference-side masking sensitivity and feature-class alignment coverage. (A) Percent of baseline aligned *A. rufa* reference sequence retained after independently masking the nonredundant dispersed-repeat union (111,377 bp; 10.89%) or MTPT union (50,774 bp; 4.97%). Query assemblies were not independently masked. (B) Coverage of annotated CDS parts and their non-CDS complement in unmasked comparisons. CDS comprises 32,700 bp; non-CDS comprises 989,759 bp and includes RNA genes and introns as well as intergenic sequence. The six primary circular-shift tests have Benjamini–Hochberg-adjusted P values of 0.001674–0.008745. The contrast concerns aligned coordinates, not conserved gene order, independent evolutionary processes, or causal effects of repeats/MTPTs.

Related response: Reviewer 1, comment 13; Reviewer 2, comments 3–5; Tables S11A–S11B.

Figure S6

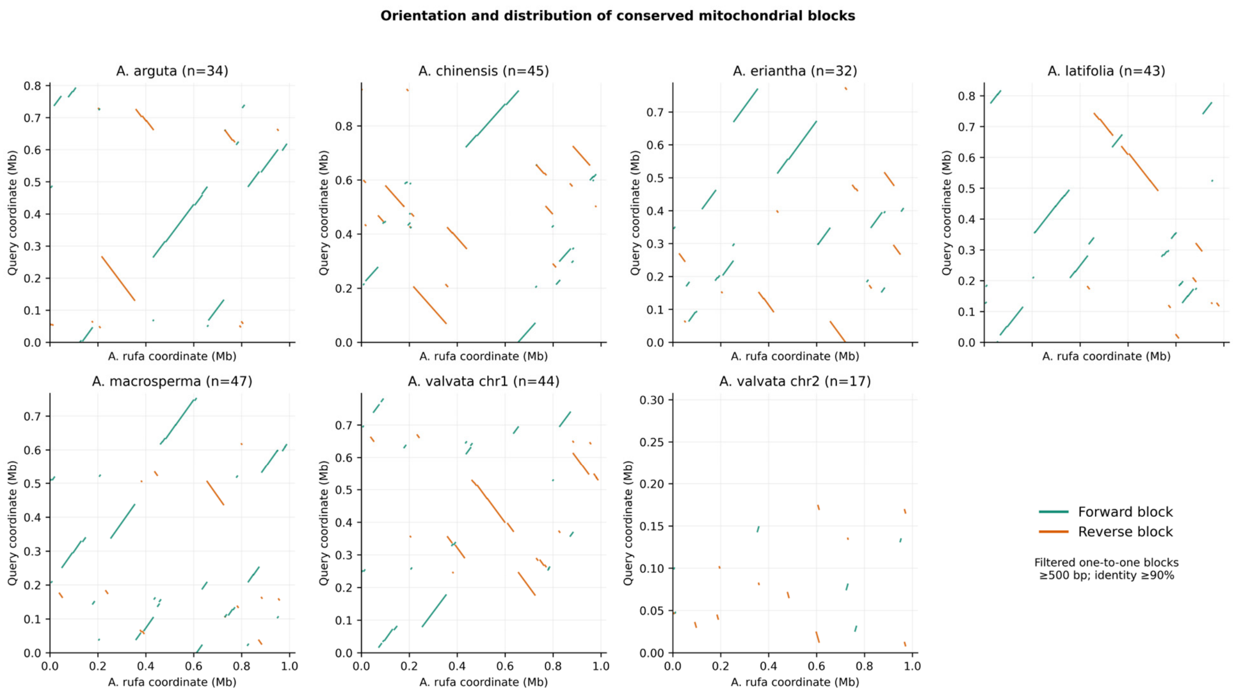

Figure S6. Component-aware dot plots of filtered one-to-one homologous mitochondrial blocks. Reference and query coordinates are shown; colors distinguish forward and reverse orientation. *A. valvata* components are displayed separately and are not artificially joined. The plotted blocks meet the 500-bp/90%-identity primary criteria. Table S10 distinguishes 174 within-component candidate discontinuities, 66 reverse intervals, and 22 component transitions excluded from breakpoint totals. Candidate coordinates may reflect evolutionary change, alternative molecular forms, or assembly representation and are not validated inversions/translocations.

Related response: Reviewer 1, comment 13; Reviewer 2, comments 1–3 and 7; Tables S9B and S10A–S10C.

Figure S7

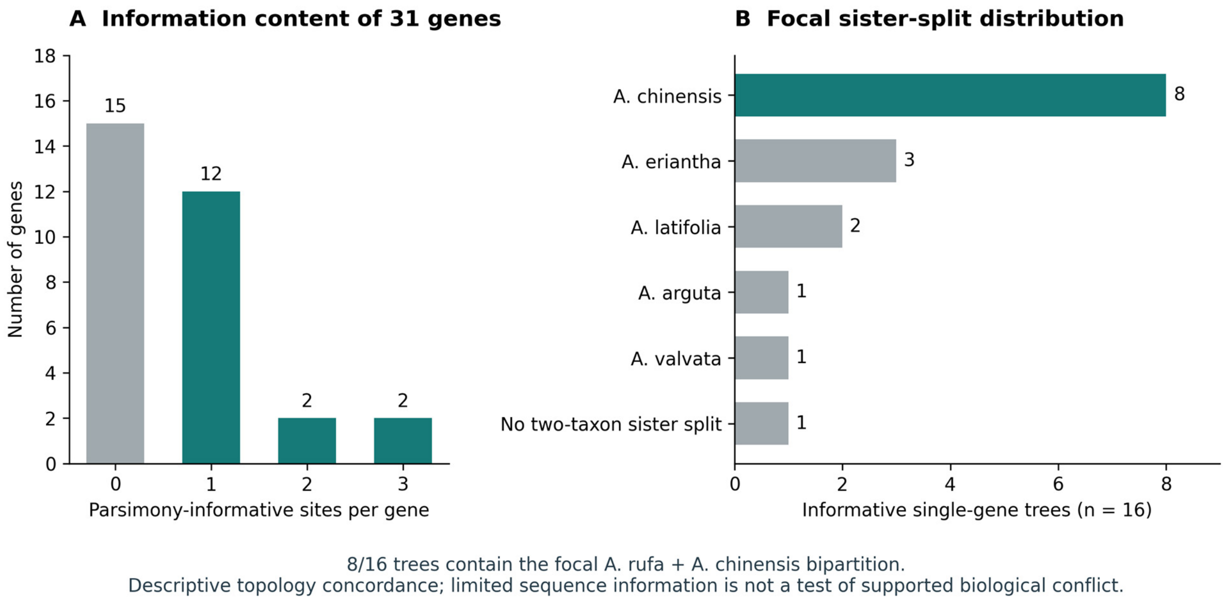

Figure S7. Information content and descriptive mitochondrial gene-tree concordance in seven *Actinidia* taxa. (A) Numbers of genes with 0, 1, 2, or 3 parsimony-informative sites: 15, 12, 2, and 2, respectively (31 genes total). (B) *A. rufa* two-taxon sister splits among the 16 informative gene trees: *A. chinensis*, 8; *A. eriantha*, 3; *A. latifolia*, 2; *A. arguta*, 1; *A. valvata*, 1; no two-taxon sister split, 1. Thus, eight informative trees contain the focal *A. rufa* + *A. chinensis* bipartition. This descriptive 50% fraction is not a formal measure of strongly supported biological conflict, and the mitochondrial topology is not a complete species tree.

Related response: Reviewer 1, comments 11–12; Tables S12A–S12B.
